# Pennsylvania State Board of Veterinary Medical Examiners, June 17, 18, 1901

**Published:** 1901-09

**Authors:** 


### PENNSYLVANIA STATE BOARD OF VETERINARY MEDICAL EXAMINERS,

June 17, 18, 1901.

As a result of the examinations held on the above-named dates, license was granted the following:

| | |
|---|---|
| Bender, Harry Ellsworth, | Lititz, Pa. (Univ. of Penna.). |
| Carlisle, Thomas S., | 8425 Germantown Ave., Chestnut Hill, Phila., Pa. (Univ. of Penna.). |
| Colton, Charles L., | Englewood, N. J. (Univ. of Penna.). |
| Gilliland, Samuel H., | 3608 Pine Street, Phila., Pa. (Univ. of Penna.). |
| Hayman, J. W., | Rohrsburg, Pa. (Univ. of Penna.). |
| Kann, R. L., | Mechanicsburg, Pa. (Univ. of Penna.). |
| McNeal, Pete, | Milton, Pa. (Ontario). |
| Morris, Franklin S., | Buckville, Pa. (New York-American). |
| Norton, Oscar M., | Aldenville, Pa. (Univ. of Penna.). |
| Olweiler, Jacob F., | Elizabethtown, Pa. (McKillip). |
| Rivers, Reuben, | North Wales, Pa. (Univ. of Penna.). |
| Stehle, Jr., Frederick, | 4272 Ridge Ave., Phila., Pa. (Univ. of Penna.). |
| Shore, C. Scott, | Lake Shore, Minn. (Univ. of Penna.). |
| Seidel, Edward, | Bryn Mawr, Pa. (Univ. of Penna.). |
| Watson, Harry Wm., | 3608 Pine Street, Phila., Pa. (Univ. of Penna.). |
| Walter, Charles R., | Conyngham, Pa. (Univ. of Pa.). |
| Woodward, Frank G., | 5912 Market St., Phila., Pa. (Univ. of Penna.). |

One applicant failed.

